# A Novel Method of Determining the Functional Effects of a Minor Genetic Modification of a Protein

**DOI:** 10.3389/fcvm.2015.00035

**Published:** 2015-11-18

**Authors:** Janhavi Nagwekar, Divya Duggal, Krishna Midde, Ryan Rich, Jingsheng Liang, Katarzyna Kazmierczak, Wenrui Huang, Rafal Fudala, Ignacy Gryczynski, Zygmunt Gryczynski, Danuta Szczesna-Cordary, Julian Borejdo

**Affiliations:** ^1^Department of Cell Biology, Center for Commercialization of Fluorescence Technologies, University of North Texas Health Science Center, Fort Worth, TX, USA; ^2^Department of Mathematics, Computer Science, and Physics, Texas Wesleyan University, Fort Worth, TX, USA; ^3^Department of Molecular and Cellular Pharmacology, Miller School of Medicine, University of Miami, Miami, FL, USA; ^4^Department of Physics and Astronomy, Texas Christian University, Fort Worth, TX, USA

**Keywords:** low expressing mutations, actin, myosin light chain, phalloidin-actin, fluorescence polarization, autocorrelation function

## Abstract

Contraction of muscles results from the ATP-coupled cyclic interactions of the myosin cross-bridges with actin filaments. Macroscopic parameters of contraction, such as maximum tension, speed of shortening, or ATPase activity, are unlikely to reveal differences between the wild-type and mutated (MUT) proteins when the level of transgenic protein expression is low. This is because macroscopic measurements are made on whole organs containing trillions of actin and myosin molecules. An average of the information collected from such a large assembly is bound to conceal any differences imposed by a small fraction of MUT molecules. To circumvent the averaging problem, the measurements were done on isolated ventricular myofibril (MF) in which thin filaments were sparsely labeled with a fluorescent dye. We isolated a single MF from a ventricle, oriented it vertically (to be able measure the orientation), and labeled 1 in 100,000 actin monomers with a fluorescent dye. We observed the fluorescence from a small confocal volume containing approximately three actin molecules. During the contraction of a ventricle actin constantly changes orientation (i.e., the transition moment of rigidly attached fluorophore fluctuates in time) because it is repetitively being “kicked” by myosin cross-bridges. An autocorrelation functions (ACFs) of these fluctuations are remarkably sensitive to the mutation of myosin. We examined the effects of Alanine to Threonine (A13T) mutation in the myosin regulatory light chain shown by population studies to cause hypertrophic cardiomyopathy. This is an appropriate example, because mutation is expressed at only 10% in the ventricles of transgenic mice. ACFs were either “Standard” (Std) (decaying monotonically in time) or “Non-standard” (NStd) (decaying irregularly). The sparse labeling of actin also allowed the measurement of the spatial distribution of actin molecules. Such distribution reflects the interaction of actin with myosin cross-bridges and is also remarkably sensitive to myosin mutation. The result showed that the A13T mutation caused 9% ACFs and 9% of spatial distributions of actin to be NStd, while the remaining 91% were Std, suggesting that the NStd performances were executed by the MUT myosin heads and that the Std performances were executed by non-MUT myosin heads. We conclude that the method explored in this study is a sensitive and valid test of the properties of low prevalence mutations in sarcomeric proteins.

## Introduction

Muscle contraction results from the ATP-coupled, cyclic interactions of the myosin cross-bridges with actin filaments. Macroscopic parameters of contraction, such as maximum tension, speed of shortening, or ATPase activity are unlikely to reveal differences between wild-type (WT) and mutated (MUT) proteins when the level of transgenic protein expression is low. This is because macroscopic measurements are done on whole organs (or on a small part of a tissue) containing trillions of actin and myosin molecules. These molecules originate from muscle fibers with different fiber structures and orientations. Moreover, molecular crowding effects are different in various parts of a ventricle. Averaging data from such a large assembly of molecules with varying orientations prevent observing minute deviations expected from mutant protein that is expressed at a low level.

**Figure 1:**
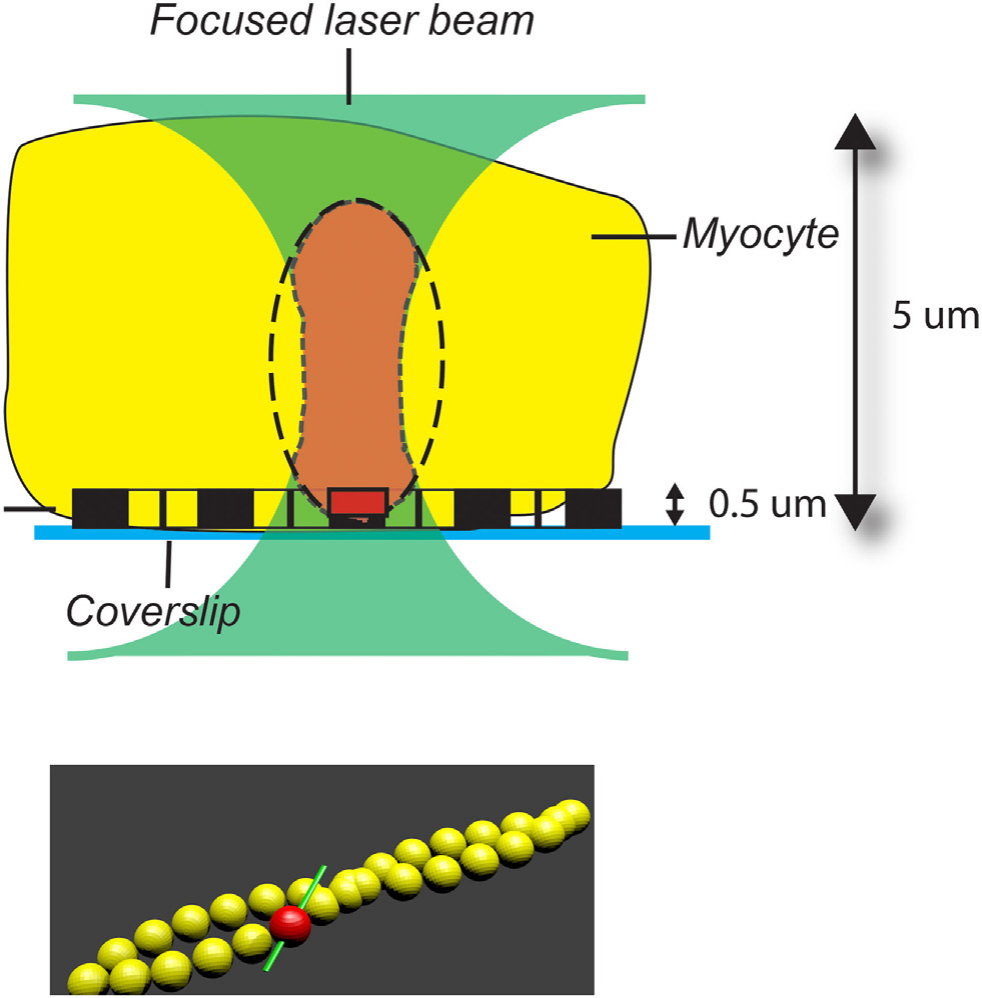
**The illustration to show advantage of using myofibrils rather than a whole ventricle or myocytes in studies when a few molecules need to be studied in *ex vivo***. Top: a laser beam (green) is focused on a myocyte. Fluorescence is collected from the region (brown) of overlap between the volume seen by a confocal microscope (ellipsoid-of-revolution, dashed line) and the laser beam. This part of a myocyte contains hundreds of myofibrils and, therefore, more than 100,000 actin molecules. If, on the other hand, only a single myofibril is studied, the fluorescence is collected only from a volume containing actin (dark brown), which contains approximately three actin molecules; Bottom: only one (red) in 100,000 actin molecules (yellow) is labeled. The transition dipole of rhodamine is green. Drawn not to-scale.

This paper suggests a novel method of circumventing the averaging problem in samples containing minor genetic modifications of myosin. The samples are analyzed by the mesoscopic method (1) (i.e., only a small number of molecules within a sample are analyzed). Observing only a small number of molecules avoids averaging data from a large assembly of molecules with varying orientations. To achieve mesoscopic conditions, only 1 out of 100,000 actin monomers in thin filaments of isolated cardiac myofibrils (MFs) was labeled. Figure 1 illustrates how the complications arising from the fact that molecules originate from muscle fibers with different structures and orientations was eliminated.

As an experimental model we used mouse ventricles that had undergone cardiac hypertrophic cardiomyopathy HCM mutation A13T (Alanine → Threonine) in the myosin regulatory light chain (RLC). This mutation was shown by population studies to cause cardiac hypertrophy with no incidences of sudden cardiac death (2–5). A13T-RLC mutation is a good choice to study the effects of low expression because in mouse hearts it is expressed at only ~10% level (6).

The conformational change of myosin heads bound to actin filaments during contraction triggers the rotation of actin protomers because thin filaments receive periodic “kicks” (momentum transfer) from the active myosin heads (7–9). As a result, the transition dipole of rhodamine fluctuates in time. We measured the parameters characterizing these fluctuations during contraction in MUT cardiac MFs from mice, and compared it with transgenic WT-RLC. Specifically, we measured the parallel (||) and perpendicular (⊥) components of the fluorescent light emitted by rhodamine-bound actin monomers and followed the fluctuations of the orientation of the transition dipole of rhodamine by computing the normalized ratio of the difference between these components, called polarized fluorescence (PF). PF is a sensitive indicator of the orientation of the transition dipole of the fluorophore (10–16). The relative fluctuation of PF depends on the number of molecules under observation (N) as √N/N (17–19). A whole papillary muscle or even a single cardiac myocyte contain a large number (>10^11^) of myosin molecules, i.e., the relative fluctuation is <10^−3%^ (i.e., only 1/100,000 of the signal carries the kinetic information). There is no hope of observing such small signal. By using a microscope capable of detecting single molecules, we were able to observe only approximately three actin molecules. For this number of molecules (*N* = 3), the relative fluctuation is √N/N = 57%. We show that the autocorrelation functions (ACF) of mesoscopic fluctuations are uniquely sensitive to the deviations from the “standard” (Std) (monotonic) decay characteristic of WT. Furthermore, the distribution of orientations of the transition dipole of rhodamine also proved to be sensitive to deviations from the Std Gaussian distribution. With the techniques used today, hypertrophy induced with only 10% penetrance of the mutation would generally go undetected. This defect is easily detected by labeling a few actin molecules and studying the kinetics and orientation changes. The results showed that the A13T mutation caused 9% ACFs and 9% of spatial distributions of actin to be non-standard (NStd; i.e., assumed a shape completely incongruous with the Std), while the remaining 91% were Std. These data suggest that the NStd performances were realized by the MUT myosin heads and that the Std performances were realized by non-MUT myosin heads. We conclude that the mesoscopic method explored in this study is a sensitive and valid test of the properties of HCM-linked mutations in myosin or its light chains that occur with low prevalence in humans and are expressed at low levels in the ventricles of animal models of HCM.

## Materials and Methods

### Chemicals, Solutions

Tetramethylrhodamine-phalloidin was purchased from Molecular Probes (Eugene, OR, USA, Cat. No. R-415). All other chemicals were from Sigma-Aldrich (St Louis, MO, USA). The composition of solutions was as in (20). Briefly, the Ca-rigor solution contained 50 mM KCl, 0.1 mM CaCl_2_, 5 mM MgCl_2_, 10 mM Tris pH 7.5, and 1 mM DTT. EDTA-rigor had the same composition except that it did not contain either Mg or Ca and contained in addition 5 mM EDTA. Relaxing solution had the same composition as Ca-rigor but contained 5 mM ATP and 2 mM EGTA. Contracting solution had the same composition as Ca-rigor but contained 5 mM ATP.

### Reconstituted Transgenic Mice Containing Human RLC

The transgenic mice were prepared by DS-C. Human WT-RLC and A13T-RLC were expressed in the mice to obtain increasing degree of reconstituted human RLC (WT or MUT A13T). Tg-WT served as control with human RLC expression of 40%. A13T mutant is incorporated in Tg mice to give 10% expression with 90% NTg.

### Preparation of Myofibrils

The investigation conforms with the Guide for the Care and Use of Laboratory Animals published by the US National Institutes of Health (NIH Publication No. 85–23, revised 1996). All animal studies were conducted in accordance with institutional guidelines (Recent Annual Animal Protocol Review, Protocol Approval # 2013/14-46-A08, approved by Dr. Michael Forster, Chairman of IACUC on 05/30/2014). Left ventricular muscle from 6-month-old Tg-A13T mice together with Tg-WT controls were shipped to Fort Worth on dry ice Kazmierczak et al. (6). Before experiments, the strips were thoroughly washed with an ice-cold EDTA-rigor solution for 0.5 h (to avoid contraction on transfer to ATP-free solution) followed by an extensive wash with Mg^2+^-rigor solution followed by the Ca^2+^-rigor solution. The muscle strips were then homogenized in Ca^2+^-rigor solution using a Heidolph Silent Crusher S homogenizer for 20 s (with a break to cool after 10 s). Freshly prepared MFs were used in each experiment.

### Labeling

Myofibrils were labeled with 0.1 nM rhodamine-phalloidin (RP) + 10 μM unlabeled-phalloidin (UP) in Ca^2+^-rigor solution as described in (21). Since the ratio of fluorescent to non-fluorescent phalloidin was 1:100,000, only 1 in 10^5^ actin monomers carried a fluorophore. UP was used to avoid uneven labeling. In the absence of UP, MFs closest to the tip of pipette that was used to add fluorophore were predominantly labeled. Phalloidin had no effect on the ATPase activity of labeled MFs: 1.33 ± 2.65 μM Pi/min/mg myofibrillar myosin without phalloidin, 1.81 ± 2.70 μM Pi/min/mg myofibrillar myosin with phalloidin. In addition, myofibrils shortened at the same speed when observed under Nomarski microscope.

### Number of Observed Molecules

**Figure 2:**
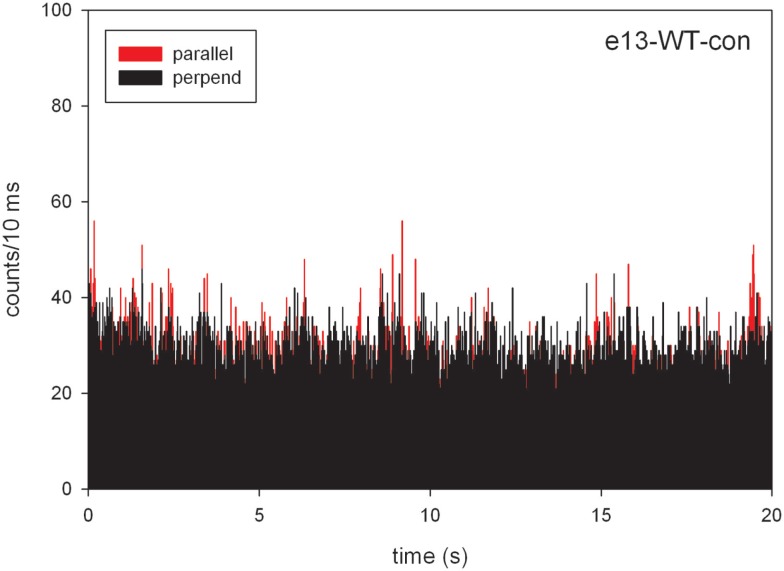
**A typical time course of polarized intensity of contracting myofibrils (MFs) from the right ventricle of WT heart**. The graph is a bar plot. The vertical scale is the number of counts during 10 ms. The intensities perpendicular (||_⊥_) and parallel (I_||_) to myofibrillar axis are black and red, respectively.

Calibration shown in supplementary Figure S1 in Supplementary Material reveals that one molecule of rhodamine resulted in the detection of 9 photons/10 ms. Figure 2 shows the intensities of the perpendicular (ch1) and parallel (ch2) channels of contracting WT MF. The average intensities were 23 and 26 counts/10 ms (note that Figure 2 is a bar plot). The number of molecules observed in one channel was 24 counts/9 count/molecule = ~2.7 molecules/10 ms/channel.

This number agrees well with the predicted number: the concentration of actin in muscle is 0.6 mM, (22) and the confocal volume is 10^−15^L. There are 3.6 × 10^5^ actin monomers in this volume. Since only 1 in 100,000 monomers are labeled, we estimate that there are approximately three to four fluorescent actin molecules in the detection volume (OV). It is important to note, however, that we only need to know that this number is small. The results must be the same whether the number is 3 (57% fluctuation) or 30 (18% fluctuation). The latter is also easily detectable by the avalanche photodiode used by our microscope.

### Cross-Linking

In contracting solution (in the presence of Ca^2+^ and ATP) MFs shorten, making it impossible to record steady-state polarization. To prevent MFs from shortening they were cross-linked with water-soluble cross-linker 1-ethyl-3-[3-(dimethylamino) -propyl] carbodiimide (EDC) (23, 24). MFs (1 mg/ml) were incubated with 20 mM EDC for 20 min at room temperature. The reaction was stopped by adding 20 mM DTT. The lack of shortening was checked by confocal microscopy after labeling MFs with 1 μM RP. Control experiments in skeletal muscle showed that cross-linking made no difference to the shape of histograms, i.e., that cross-bridges cycled normally) (23, 24). Phalloidin had no effect on the ATPase activity of EDC cross-linked MFs: it was 2.44 ± 0.58 μM Pi/min/mg myofibrillar myosin without phalloidin and 2.33 ± 1.35 μM Pi/min/mg myofibrillar myosin with phalloidin.

### Sample Preparation

Cardiac MFs attached weakly to the glass and were easily displaced by washing. To help them attach strongly, the coverslips were thoroughly cleaned with 100% ethanol, and spin coated with Poly-l-lysine solution (Sigma-Aldrich 0.1%) at 3,000 rpm for 120 s using a spincoater P6700 (Specialty Coating Systems, Indianapolis, IN, USA).

### Probability Distribution Measurements

The measurements were done on an MT200 confocal system (PicoQuant, Berlin, Germany) coupled to an Olympus IX 71 microscope. The 2-M data points were collected every 10 μs for 20 s and smoothed by binning 1,000 points together. Before each experiment, the instrument was optimized with 50 nM solution of long fluorescent lifetime dye (rhodamine 700), making sure that the geometrical correction factor G (ratio of the orthogonal components) was the same as measured in the specialized instrument (PicoQuant FT 200). The excitation was by a 532 nm CW laser. To ensure that the exciting light was strictly linearly polarized, a polarizer was inserted before the entrance of the light to the microscope. The laser polarization was vertical on the microscope stage. The laser power incident on a sample was 100 μW. The confocal aperture was 50 μm and magnification of the objective was 60× (NA = 1.2). The emitted (fluorescent) light was split 50/50 by a birefringent prism and each component was detected by a separate avalanche photodiode (APD). The || and ⊥ (with respect to the laboratory frame of reference) analyzers were inserted before each APD. Channels 1 and 2 measured the polarized intensities oriented ⊥ and || to the myofibrillar axis, respectively. MFs were always placed with the axis pointing vertically on the microscope stage. Adopting the notation of (12), _||_I_||_ (recorded in ch2) is the polarized intensity obtained with the exciting light polarized parallel to muscle axis and detected through analyzer oriented parallel to the axis, and _||_I_⊥_ (recorded in ch1) is the polarized intensity obtained with exciting light polarized parallel to muscle axis and detected through the analyzer oriented perpendicular to the axis. We, thus, measured _||_I_⊥_ (ch1) and _||_I_||_ (ch2). We calculated parallel polarization of fluorescence as PF_||_ = (_||_I_||_ − _||_I_⊥_)/(_||_I_||_ + _||_I_⊥_) = (ch2 − ch1)/(ch2 + ch1).

### Time Resolved Anisotropy

Fluorescence anisotropy was measured by the time-domain technique using FluoTime 200 fluorometer (PicoQuant, Inc.). The excitation was by a 532-nm laser pulsed diode, and the observation was through a monochromator at 590 nm with a supporting 590-nm-long wave pass filter. The FWHM of pulse response function was 370 ps. Time resolution was better than 10 ps. The intensity decays were analyzed in terms of a multi-exponential model using FluoFit software (PicoQuant, Inc.). All experiments were performed at room temperature (~23°C).

### Anisotropy Decay

To ascertain whether the phalloidin probe is immobilized by the protein so that the transition dipole of the fluorophore reflects the orientation of the protein, we measured the decay of anisotropy, defined as *r* = (I_||_ − I_⊥_)/(I_||_ + 2I_⊥_) using green 532 nm excitation. We first measured the fluorescence lifetime of RP (100 nM) in rigor solution (not shown). About 92.19% of the intensity decay could be fitted by a single exponential function convolved with the instrument response function, giving the intensity weighted lifetime of 2.453 ns. The decay of anisotropy of RP alone was fitted by a single exponential function *r*(*t*) = *R*_∞_ + *R*_o_ × exp(−t/θ_1_) where *R*_∞_ was the value of anisotropy at infinite time, *R*_o_ was the amplitude of the anisotropy at time 0, and θ_1_ was the rotational correlation time. The best fit gave *R*_∞_ = 0, *R*_o_ = 0.241, and a correlation time θ_1_ = 0.703 ns (black circles, Figure S2 in Supplementary Material). The decay of anisotropy of RP + MFs (red circles) was fitted by a sum of two exponential functions *r*(*t*) = *R*_∞_ + *R*_1_ × exp(−*t*/θ_1_) + *R*_2_ × exp(−t/θ_2_) with the best fit giving *R*_∞_ = 0.121, *R*_o_ = *R*_1_ + *R*_2_ + *R*_∞_ = 0.258, a long correlation time θ_1_ = 2.040 ms, and a short correlation time θ_2_ = 0.443 ns. The long correlation time (82.15% of the total) was due to the rotation of rhodamine immobilized by actin filaments and a short correlation time (17.85% of total) was due to the rotation of mobile phalloidin, consistent with the rotation of a molecule with *M*_w_ = 1,250. Thus, the mobile fraction was contributed by the minority of fluorophores and we conclude that RP labeling is appropriate for measuring polarization of fluorescence in muscle.

### Statistical Analysis

It was carried out using Systat software (SigmaPlot 11.02 and Origin v. 8.5, Northampton, MA, USA). Goodness of fit was assessed by SE or reduced (χ^2^) and by adjusted coefficient of determination (AR^2^). The distribution functions were fitted by 3-parameter Gaussian function.

## Results

### Mesoscopic Measurements

**Figure 3:**
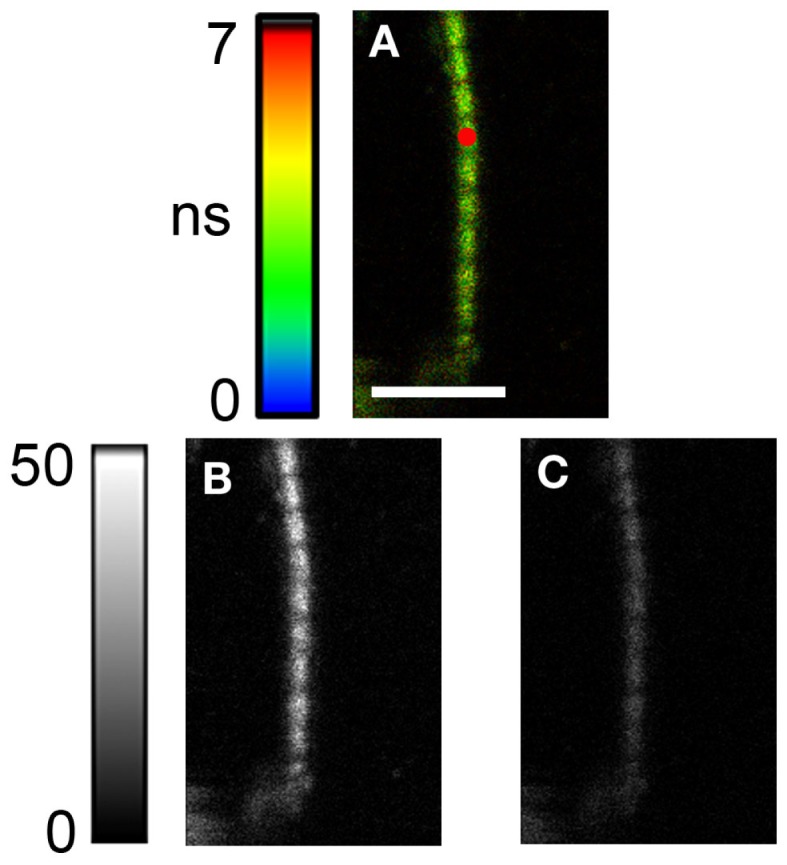
**Image of A13T myofibril in rigor labeled with rhodamine-phalloidin**. **(A)** FLIM (Fluorescent Lifetime Image), **(B)** parallel, and **(C)** and perpendicular intensity images of a A13T myofibril in rigor. Lifetime scale, in nanosecond, is at left of **(A)**. The intensity scale [left of **(B,C)**] is in arbitrary units with 0 corresponding to white and 255 to black. Notice that muscle in rigor is very anisotropic, because **(B)** is more intense than **(C)**. By contrast, contracting muscle is isotropic (Figure 1). The red circle is the projection of the confocal aperture on the sample plane (diameter is 50/60 = 0.84 μm). Images were acquired with the PicoQuant Micro Time 200 confocal lifetime microscope. Excitation with a 470 nm pulsed laser, emission through LP500 filter. Sarcomere length 3.0 μm. Scale bar in A is 5 μm.

Measurements were done on MFs, as illustrated in Figure 3. The MF was labeled with 0.1 nM RP + 10 μM UP. In contrast to skeletal muscle, where nebulin prevents phalloidin from labeling all-but the pointed ends of actin filaments (25, 26), in the nebulin-free heart muscle the entire I-bands are labeled. The dark areas do not contain actin (H-bands). The red circle is a 2D projection of the confocal aperture on the image plane. Its diameter (0.84 μm) is equal to the diameter of the confocal aperture (50 μm) divided by the magnification of the objective (60×). As expected from the aligned array of polar actin filaments (8), the parallel (I_||_) and the perpendicular (I_⊥_) polarization images (B and C) have different intensities, indicating that the fluorescence is highly anisotropic. The fluorescence signal was collected through the pinhole (red circle) after the initial photobleaching. We estimated in Section “Materials and Methods” that approximately three actin molecules contributed to this signal.

### Contraction Kinetics

**Figure 4:**
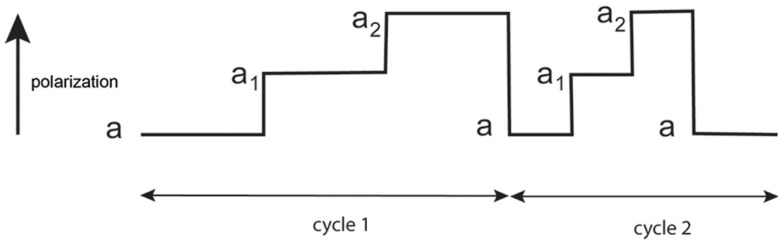
**Schematic representation of changes of polarized fluorescence of transition dipole of rhodamine bound to actin**. Note that duration of each intermediate state is random. a1, a2, and a3 are the values of steady-state anisotropy determined from anisotropy decay curves such as shown in Figure S2 in Supplementary Material. Anisotropy of actin is initially low because it is not immobilized by a cross-bridge (XB). It subsequently increases because actin rotation is now restricted by a weakly bound myosin heads. It then further increases because actin rotation is restricted by isomerization of the myosin head (power stroke) that causes strong binding to actin.

The orientation of the transition dipole of rhodamine embedded in actin protomer changes constantly during contraction because during acto-myosin interactions, actin is being constantly “kicked” by the myosin heads. The manifestation of these kicks is seen in PF changes (or changes of anisotropy) as occurring in three stages: (1) when myosin heads bind to thin filaments, (2) when isomerization of bound myosin heads occur, and (3) when myosin heads dissociate from thin filaments. These steps are schematically illustrated in Figure 4.

**Figure 5:**
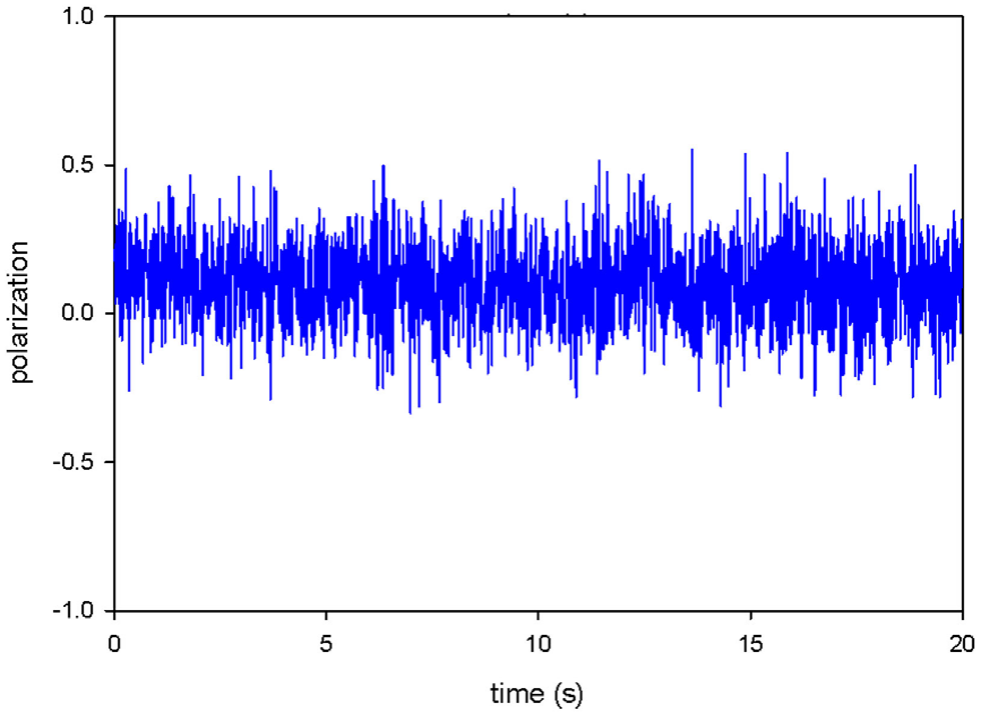
**Actual fluctuations of polarized fluorescence of phalloidin transition dipole in contracting myofibril**. The overall signal has characteristics of a random Gaussian signal.

Figure 5 shows the time course of PF from the actual experiment. It results from summation of individual fluctuations (like the ones shown schematically in Figure 4) during 20-s experiment.

Fluctuations are characterized by an ACF(τ) (17, 18). The rate of decay of ACF from the maximal value to 0 is a reflection of how quickly the average fluctuation crosses the equilibrium baseline (19). To extract the rate constants from the time course, such as shown in Figure 5, we solved the differential equations (27) describing the ACF of a three-state process. The resulting ACF determines the rates of *a*_1_ → *a*_2_, *a*_2_ → *a*_3_, and *a*_3_ → *a*_1_ transitions (rates *k*_1_–*k*_3_). Note, however, that all experimental ACFs were similar to two-parameter hyperbolic decay, i.e., we were able to define only two kinetic constants. This is due to the fact that the rate of cross-bridge binding to actin is very rapid (28) and the time resolution of the present method is 10 ms. Thus, the rapid process (*k*_1_) was undetectable. We, therefore, report only *k*_2_ (the rate of cross-bridge isomerization or power stroke) and k_3_ (the rate of cross-bridge detachment from thin filaments).

### Kinetics of Orientation Change During Contraction

**Figure 6:**
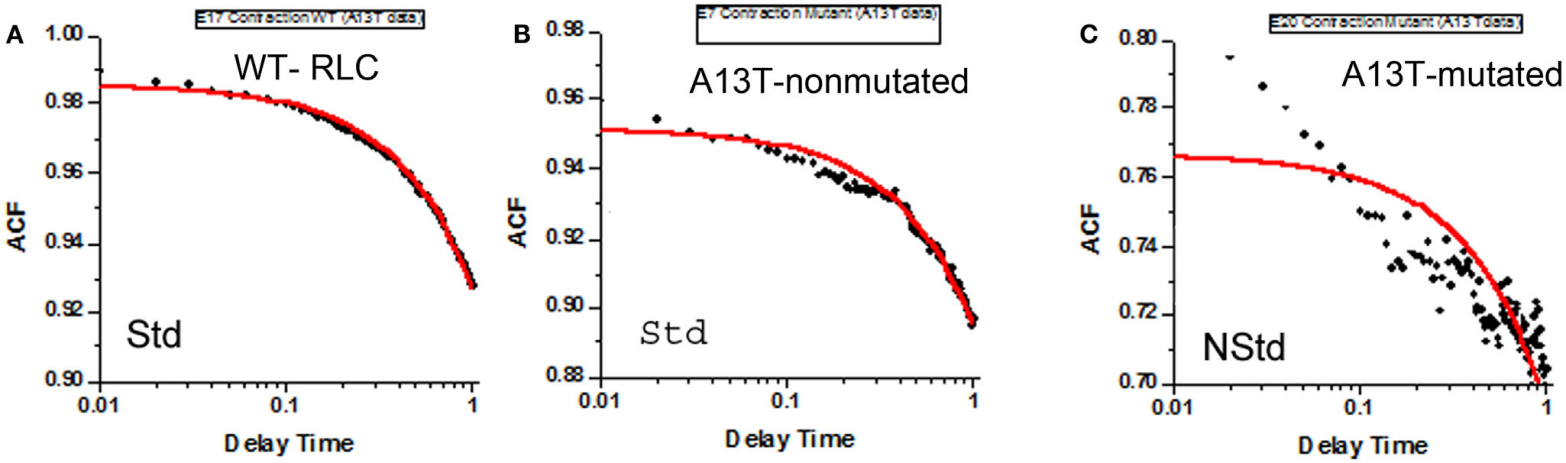
**Representative trace of normalized autocorrelation function of polarization of fluorescence**. **(A)** Data are from 33 contracting sarcomeres prepared from the left ventricle of Tg-WT. Traces are labeled Std for Standard (based on adjusted coefficient of determination, see text). The fact that the correlation decays in time in this figure, and Figures 6B,C indicate that the orientation of absorption/emission dipoles change in time. The fact that ACF decays to a value >0 is due to the fact that mean polarization was non-zero. Delay time is in seconds. All 33 experiments are shown in Figure S3 in Supplementary Material. **(B)** Representative trace of normalized autocorrelation function of polarization of fluorescence of contracting sarcomeres prepared from the left ventricle of Tg-A13T mice which apparently was non-transfected (labeled “Std” for Standard. All 33 experiments are shown in Figure S4 in Supplementary Material. **(C)** Representative trace of normalized autocorrelation function of polarization of fluorescence of a contracting myofibril prepared from the left ventricle of Tg-A13T mice expressing ~10% of transgene (labeled “NStd” for non-standard. All traces of normalized autocorrelation functions of Tg-A13T mouse myofibrils are shown in Figure S4 in Supplementary Material.

We first measured the correlation functions of contracting Tg-WT MFs. Figure 6A shows an example of the ACF functions out of the total of 33 experiments. As shown in Kazmierczak et al. (6), WT L4 hearts expressed 40% transgene (human ventricular RLC) and were 60% NTg in comparison to 10% A13T: 90% NTg in A13T hearts. The fit of Std WT MFs to ACF was excellent and we have not observed any NStd populations. This result suggests that WT and NTg XBs present in WT hearts were not kinetically different.

The correlation function could be classified as “Std” and “NStd.” To quantify the difference between them, we computed the adjusted coefficient of determinations (AR^2^) of the non-linear fit to the ACF. AR^2^ is a good measure of goodness of fit because it takes into account scaling of the dependent variable. The closer AR^2^ is to the value of 1, the better the fit. ACFs shown in Figures 6A,B had AR^2^ of 0.90 and 0.93, respectively, and, thus, they were classified as Std.

The quantitative comparison of the kinetics of Std WT and Std A13T correlation functions is shown in Table 1. There was no statistically significant difference between either parameter.

**Table 1:** **The kinetic constants of contracting WT and A13T-mutated left ventricular muscle**.

Muscle	*k*_2_ (s^−1^)	*k*_3_ (s^−1^)
WT-RLC (standard)	1.03 ± 0.72	0.09 ± 0.09
A13T-non-mutated (standard)	1.45 ± 0.76	0.19 ± 0.45

*The errors are SD of 30 experiments. The non-standard data have been excluded from the total Tg-A13T data*.

The −0.355 difference in k_2_ was statistically *insignificant* (*t* = −1.954, *P* = 0.056) with 57 degrees of freedom. Ninety-five percent confidence interval for difference of means was −0.721 to 0.00880. The −0.101 difference in k_3_ was statistically *insignificant* (*t* = −1.214, *P* = 0.229) with 60 degrees of freedom. Ninety-five percent confidence interval for difference of means was −0.267 to 0.0654. This supports the conclusion that the Std data from MUT A13T ventricle represent the behavior of NTg XBs in Tg-A13T muscles.

We next analyzed NStd ACFs. There were no NStd ACFs of WT muscle. As shown in Figure S4 in Supplementary Material, 3 (possibly 5) out of 33 experiments on A13T sarcomeres showed NStd ACFs. Examples of ACFs are shown in Figure 6C. ACFs had AR^2^ of 0.36 and 0.30 and, thus, they were classified as NStd. The average ± SD of ACFs of all Std fits was 0.88 ± 0.07, whereas average of “NStd” fits was 0.33 ± 0.03.

No meaningful fit to the ACFs of NStd A13T muscles could be made. The quantitative comparison of the kinetics of Std A13Tand NStd A13T correlation functions was impossible. (For example, SD was large, 64% of the mean). Table 2 shows the kinetic constants of Std ACFs only.

**Table 2:** **Top row: mean *k*_2_ and *k*_3_ of standard fit**.

Muscle	*k*_2_ (s^−1^)	*k*_3_ (s^−1^)
A13T-non-mutated (standard)	1.45 ± 0.76	0.19 ± 0.45
A13T-mutated (non-standard)	–	–

*They are the same as Std Tg-A13T of Table 1. Bottom row, no meaningful fit to the ACFs of non-standard A13T muscles could be obtained. Errors are SD*.

### Steady-State Distribution of Orientations During Contraction

**Figure 7:**
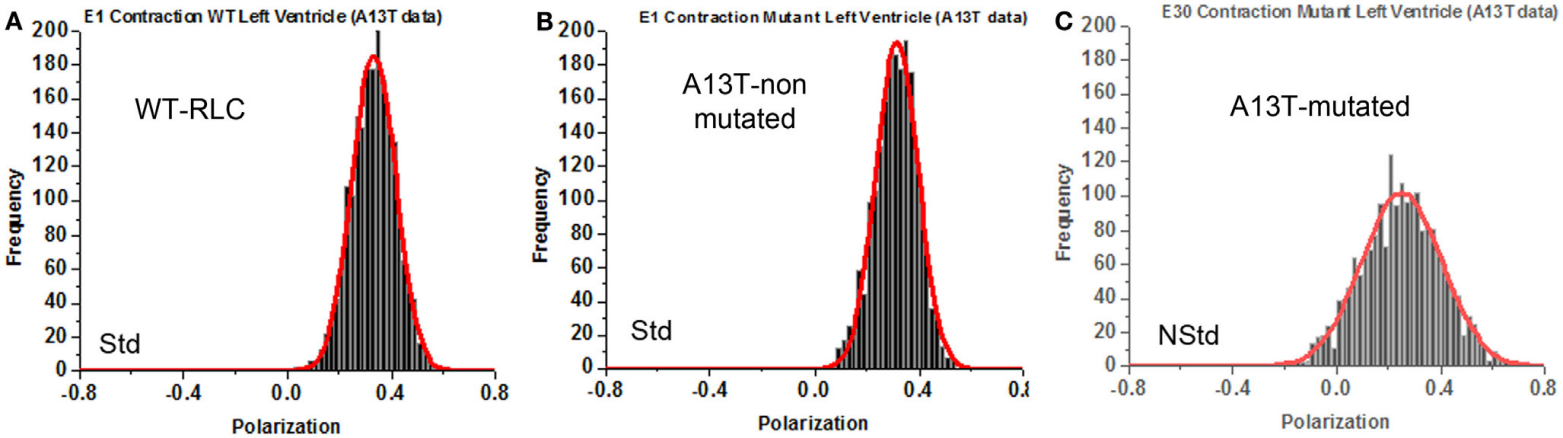
**Representative histogram of histograms of polarization of fluorescence of contracting sarcomeres prepared from left ventricle of mice**. **(A)** Data from 33 experiments from WT ventricle mice that were classified as “standard.” **(B)** Representative histogram of 30 histograms of polarization of fluorescence of contracting sarcomeres prepared from the left ventricles of Tg-A13T mice that were classified as “standard.” These muscles were negative for the A13T mutation. **(C)** Representative histogram of polarization of fluorescence of contracting sarcomeres prepared from the left ventricles of Tg-A13T mice that were positive for mutation (labeled “NStd” for non-standard.

A histogram is a plot of the number of times a given polarization of fluorescence that occurred during 20 s contraction, plotted against the value of polarization of fluorescence. Figure 7A shows an example of “Std” histogram of PF selected from a pool of 33 Std histograms of contracting WT MFs. Figure 7B shows an example of the histogram of PF selected from 30 histograms of contracting A13T MFs that could be classified as “Std.” Figure 7C shows an example of a histogram of PF of contracting A13T MFs selected from three histograms that could be classified as “NStd.” The histograms were fitted with a Gaussian curve *y* = *a* × exp[−0.5(*x* − *x*_o_/*b*)^2^]. All the histograms of Tg-WT mice are shown in Figure S5 in Supplementary Material. All the histograms of Tg-A13T mouse are shown in Figure S6 in Supplementary Material. All the histograms of Tg-A13T mice are shown in color in Figure S6 in Supplementary Material.

A histogram can be quantitatively characterized by the values of skewness and kurtosis. A positive skewness means that the tail of the curve points toward positive values of the histogram (20). A negative skewness means that the tail of the curve points toward the negative values of the histogram. A positive kurtosis is expressed by long tails and lower peaks compared to the Gaussian curves. A negative kurtosis means that the tails are smaller and the peaks are taller than those of Gaussian curves. Of the 33 experiments carried out on WT-RLC MFs (Figure S5 in Supplementary Material) all showed Std behavior. Of the 33 experiments on A13T MFs (Figure S6 in Supplementary Material), 30 showed Std behavior and 3 experiments (9%) showed NStd behavior. These three experiments were quantitatively different to the remaining 30 curves. It showed different histogram characteristics. These characteristic parameters (peak polarizations, skewness, and kurtosis) of a Gaussian curve are summarized in Table 3. It compares parameters of the 33 WT-RLC and 30 histograms of the A13T-non-MUT Std only data (three NStd experiments were excluded).

**Table 3:** **Polarization values of 33 WT-RLC and 30 A13T-non-mutated and 3 A13T-mutated myofibrils from the left ventricle of contracting heart**.

Muscle	Mean polarization	Skewness	Kurtosis*
WT-RLC (standard)	0.321 ± 0.033	2.886 ± 0.574	7.749 ± 3.734
A13T-non-mutated (standard)	0.314 ± 0.028	2.383 ± 0.260	4.610 ± 1.259
A13T-mutated (non-standard)	0.250 ± 0.037	1.623 ± 0.262	1.450 ± 1.021

* indicates that the change was significant at 5% level.

The 0.00695 difference in the mean polarization was statistically insignificant (*t* = 0.882, *P* = 0.381) with 61 degrees of freedom. The 95% confidence interval for the difference of means was −0.00880 to 0.0227. Surprisingly, the 0.502 difference in skewness was statistically significant (*t* = 4.444, *P* < 0.001) with 61 degrees of freedom. The 95% confidence interval for the difference of means was 0.276–0.728. Similarly, the 3.135 difference in kurtosis was statistically significant (*t* = 4.434, *P* < 001) with 61 degrees of freedom. The 95% confidence interval for difference of means was 1.721–4.548. These results suggest that the very process of making transgenic animals produces some, albeit minor, differences in the distribution and suggests that Std histograms were contributed by ventricles that did not undergo A13T exchange.

Table 3 also shows comparison between non-MUT and A13T-MUT MFs. The −0.064 difference in the mean polarization was statistically highly significant (*t* = −4.008, *P* < 0.001) with 31 degrees of freedom. The 95 percent confidence interval for difference of means was −0.0994 to −0.0324. The 3.160 difference in kurtosis was statistically highly significant (*t* = −4.926, *P* < 0.001) with 31 degrees of freedom. The 95 percent confidence interval for difference of means was −4.631 to −1.919. The −0.760 difference in skewness was statistically highly significant (*t* = −5.967, *P* < 0.001) with 31 degrees of freedom. The 95 percent confidence interval for difference of means was −0.057 to −0.518. These results show that the NStd histograms differed very significantly from Std ones and suggest that NStd histograms represented the ~10% MUT A13T preparations. No NStd histograms were observed for WT muscles.

## Discussion

The method described in this report has several advantages compared with the method where a large number of molecules are observed. It avoids signal averaging, which would have resulted in perfect Gaussian distributions, no matter which sample population was observed (due to Central Limit Theorem of statistics). It would have caused loss of equilibrium kinetic rates. [Kinetic rates of a large assembly of molecules can only be obtained when equilibrium is disturbed (29, 30)].

A method that relies on the measurements of a few molecules of actin can readily distinguish between the MUT vs. non-MUT molecules by observing changes in the orientation of the transition dipole of a few actin-bound rhodamine fluorophores. Since the dye is firmly attached to actin, the orientation changes are due to the fact that during muscle activity actin receives the periodic “kicks” from myosin heads. The number of these kicks is small: actin monomers in a thin filaments form a highly cooperative units that are connected by a tropomyosin double helix (7, 31–35). Each unit will interact with the myosin cross-bridge at least 100 times during our experiment lasting 20 s. This number is arrived at as follows: there are ~362 actin monomers and 180 myosin heads in one actin filament. ATPase activity of myosin is ~4/s/myosin head. The duty ratio of myosin is 20% (i.e., only 20% of myosins interact with actin during contraction). Therefore, 180/5 = 36 myosins interact with one actin filament ~2,880 times in 20 s. Each actin filament is ~1 μ (1,000 nm) long, and each cooperative unit is 38.5 nm long. There are 1,000/38.5 = 26 cooperative units in an actin filament. Each cooperative unit, thus, interacts with myosin head ~2,880/26 = 110 (~100) times/20 s. Because actin is labeled sparsely, most of the units do not contain a fluorescent dye, and, therefore, most of the interactions will not be registered by the photodiode. But once in a while, XBs will encounter the unit that contains the fluorophore, and this interaction will be registered. This number will be small, leading to a large fluctuation (57%). If mutations in the RLC make a difference to this interaction, 1 out of 10 interactions, like in A13T muscle, will be NStd. This means that at least 10 experiments are necessary to have a reasonable chance of detecting the temporal effect of the A13T mutation. Indeed, we observed NStd behavior of actin in 9% out of 33 experiments.

The comparison of Std WT and Std A13T data showed no difference in kinetic constants (Table 1), indicating similar behavior of NTg XBs (present in 60 and 90% in Tg-WT) and Tg-A13T muscles, respectively. Since the fit of the MUT muscle excluded three highly NStd sets of data, the remaining 30 experiments for A13T (Std) showed no significant differences compared with WT (Table 1). The fit of Std A13T MFs to the ACF was slightly poorer than fit of WT data in spite of exclusion of highly NStd experimental curves (Figures 6A,B). Even though muscle had high content of NTg XB, we expected no significant differences between the kinetics of WT and A13T preparations. This expectation was met (Table 1) and is supported by physiological data on Tg-WT and NTg muscle fibers (6).

Figure 6 shows that there is a large qualitative difference between the cross-bridge behavior of the MUT and WT cross-bridges. Even though the differences could not be quantified, because of a poor fit to NStd correlation functions, the difference is obvious by inspection. Interestingly, even though the A13T mutation is sparsely expressed in the A13T myocardium, it triggered macroscopic changes in the heart morphology manifested by cardiac fibrosis and the functional changes, such as significantly enhanced contractile force and reduced actin-activated myosin ATPase activity, are compared to NTg/WT hearts (6). Based on these obvious macroscopic changes, it was speculated that this particular HCM-causing mutation works most likely through a “poison-peptide” mechanism (6).

The RLC-A13T mutation that was chosen here as a model is one of the mutations of the sarcomeric proteins associated with familial hypertrophic cardiomyopathy (FHC) (3, 36). FHC is an autosomal-dominant disease, characterized by left ventricular hypertrophy, myofibrillar disarray, and sudden death. It is caused by missense mutations in various genes that encode for β-myosin heavy chain (37), myosin-binding protein C (38), ventricular RLC, and essential light chain (3, 36). The MUT proteins are rarely expressed at 100% level, but A13T mutation is an extreme example of very low expression. The A13T mutation is located near the phosphorylation site (Ser-15) of the human cardiac RLC. Even though it was expressed only at 10% level, it had threefold lower *K*_Ca_ than wild-type light chain (39). The phosphorylation of this mutant increased the Ca^2+^ affinity sixfold (39). Our method of detecting such a low level of expression may find application in a diagnostic laboratory. All that is needed is a confocal microscope equipped with a polarization optics and a laser. The method described here is highly sensitive. Its validity in the mutation of myosin RLC has been tested, but it may be applicable also in studies of other genetic mutations expressed in mouse myocardium at a very low levels. It seems reasonable to use this new approach for re-analyzing already existing models that develop HCM. It would be even more applicable in studies of a disease causing mutation that may work through other mechanisms (3, 40, 41). Finding the mechanisms of heart failure due to HCM mutations is clinically important not only for patients with HCM who have the A13T mutation or other RLC mutations, but also for other patients with mutations elsewhere in the sarcomere (41). Since reverting HCM-causing mutations to their WT state is not currently possible, revealing specific disease mechanisms may lead to identification of protein-specific therapeutic targets that could prevent disease progression and improve quality of life of HCM patients (42). Due to the high sensitivity of the method described here, it may be applicable in studies of a wide range of cardiomyopathy causing mutations that are expressed at various levels in animal models of heart disease.

The most challenging part of the technique is the detection of the weak fluorescent signal. Because measurements are done *ex vivo*, the contribution of the background to the total signal is considerable. Since autofluorescence decreases at higher wavelengths (43), the choice of a red dye, rhodamine, is a necessary one. In the future, we propose to use even redder dye, rhodamine633, which has an additional advantage that it can be excited with an inexpensive 633 nm laser (or even with a red laser pointer).

It should be noted that labeling of actin has significant advantages over labeling cross-bridges. First, it is mild, involving only addition of RP under physiological conditions, whereas labeling of the cross-bridges involves incubation under extreme conditions (37°C, in the presence of anti-schizophrenia drug trifluoperazine) (44). Second, labeling actin is very quantitative. The ratio of fluorescent to non-fluorescent phalloidin determines exactly the fraction of actin monomers that are labeled. Even though the bond between actin and phalloidin is non-covalent, it is strong enough to make rhodamine firmly fixed with respect to the thin filament axis [our anisotropy decay measurements (Figure S2 in Supplementary Material) are in line with earlier measurements].

## Conflict of Interest Statement

The authors declare that the research was conducted in the absence of any commercial or financial relationships that could be construed as a potential conflict of interest.

## Abbreviations

A13T, alanine to threonine substitution in myosin RLC; A13T-RLC, myosin regulatory light chain containing the alanine to threonine A13T mutation; A13T-mutated, part of cross-bridges that did undergo A13T Myosin RLC mutation; A13T-non-mutated, part of cross-bridges that did not undergo A13T Myosin RLC mutation; APD, Avalanche PhotoDiode; ACF, AutoCorrelation FunctionEDC, 1-ethyl-3-[3-(dimethylamino)propyl]carbodiimide; FCS, Fluorescence Correlation Spectroscopy; MF, Myofibril(s); NTg, non-transgenic (mouse RLC); OV, Observational Volume = Confocal Volume; PF, polarization of fluorescence; RLC, regulatory light chain of myosin; RP, phalloidin labeled with TMRIA; TMRIA, tetramethylrhodamine-5-iodoacetamide dihydroiodide (5-TMRIA) (single isomer); Tg, transgenic (human RLC); UP, unlabeled phalloidin; WT, wild type = WT-RLC; XB, cross-bridge.
