# Supplementary material for: A Novel Method of Determining the Functional Effects of a Minor Genetic Modification of a Protein
Source: Front Cardiovasc Med. 2015 Nov 18;2:35. doi: 10.3389/fcvm.2015.00035 (PMC4671333; doi:10.3389/fcvm.2015.00035)
Supplement: Supplementary file 1 [file data_sheet_1.pdf]

SUPPLEMENTAL DATA

Supplemental Figure 1S.

Number of observed molecules. It is essential to make sure that this number of observed molecules is known precisely and that it be small. To measure this number we need to compare the rate at which photons are arriving from muscle with the rate at which photons are arriving from a single molecule of rhodamine. To this end we measured number of free TMRIA molecules diffusing in and out of the confocal volume of our microscope. We then systematically decreased the number of observed molecules and recorded the associated signal intensity. To determine the number of observed molecules we measured a autocorrelation function (ACF) by the Fluorescence Correlation Spectroscopy (FCS). The value of the correlation function at a delay time 0 [G(0)] is equal to the inverse of the number of molecules N contributing to the signal, N=1/G(0) [1, 2]. Correlation functions were obtained for the solution of TMRIA (tetramethylrhodamine-5-iodoacetamide dihydroiodide) in the range 50-0.5 nM. An example of the autocorrelation function obtained at a concentration of 5.1 nM is shown in **Fig. 1SA**. The G(0) was 0.11, giving the number of molecules ~9. **Fig. 1SB** shows the amplitude of the signal plotted against the average number of molecules in the DV obtained by FCS. We were unable to measure autocorrelation functions at low dye concentrations (<5.1 nM TMRIA) so the curve needed to be extrapolated to a single molecule. Extrapolation was by an exponential growth, single exponent, 2 parameters fit $y=ab^x$. The fit revealed that the number of photons contributed by 1 molecule was 9/10 ms or 900 counts/s per channel.

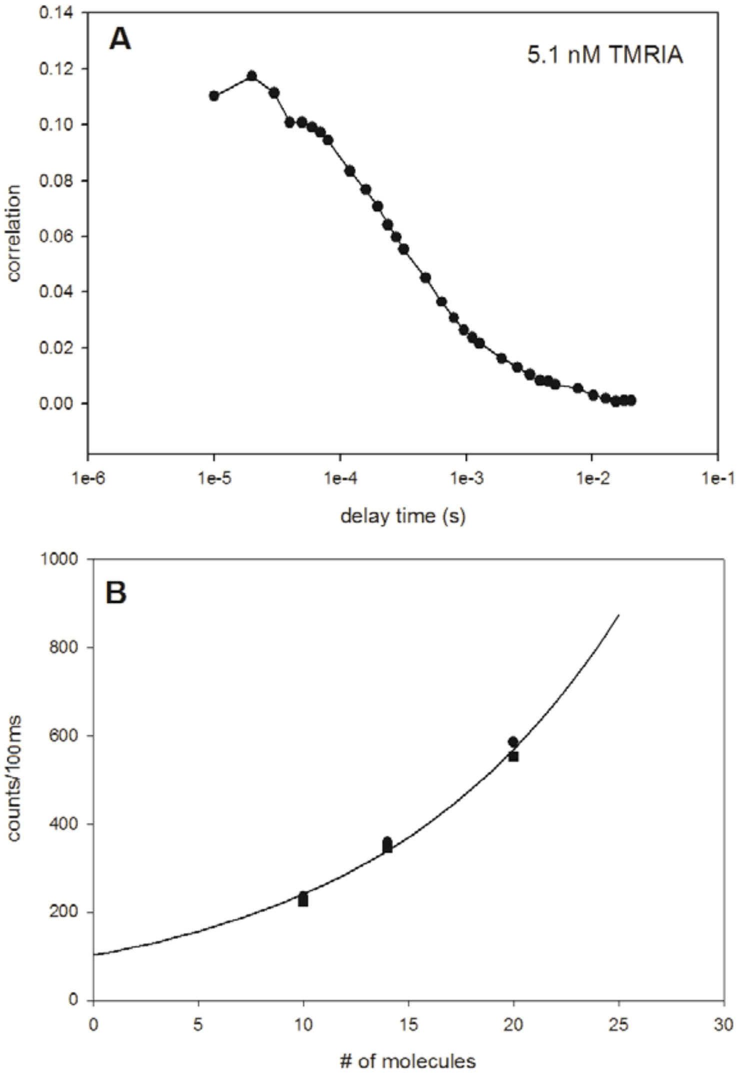

Supplemental Figure 2S.

Decay of anisotropy of 0.1 μM RP alone (black) and of 0.1 μM RP+0.5 mg/mL Tg-WT myofibrils (red).

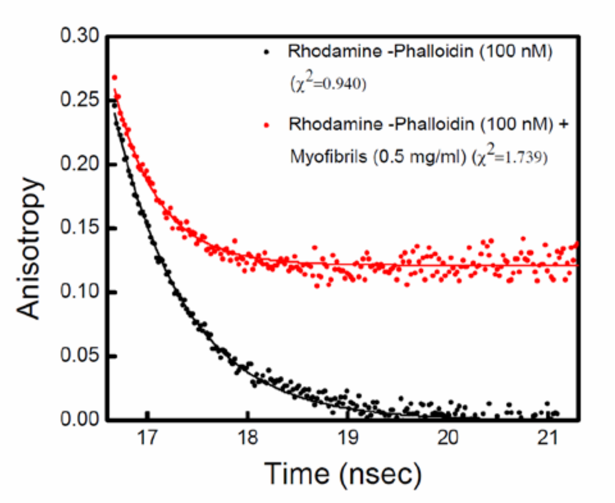

Supplemental Fig. 3S. Normalized autocorrelation functions of all 33 experiments of polarization of fluorescence of contracting myofibril prepared from the left ventricle of **Tg-WT** mouse. Circles are experimental data. A red line is the fit to the analytical solution for ACF (equation of Fig. 3S). The fact that the correlation decays in time indicates that the orientation of absorption/emission dipoles change in time. The fact that ACF decays to a value >0 is due to the fact that mean polarization was non-zero. Delay time is in seconds. There are no nonstandard ACF's.

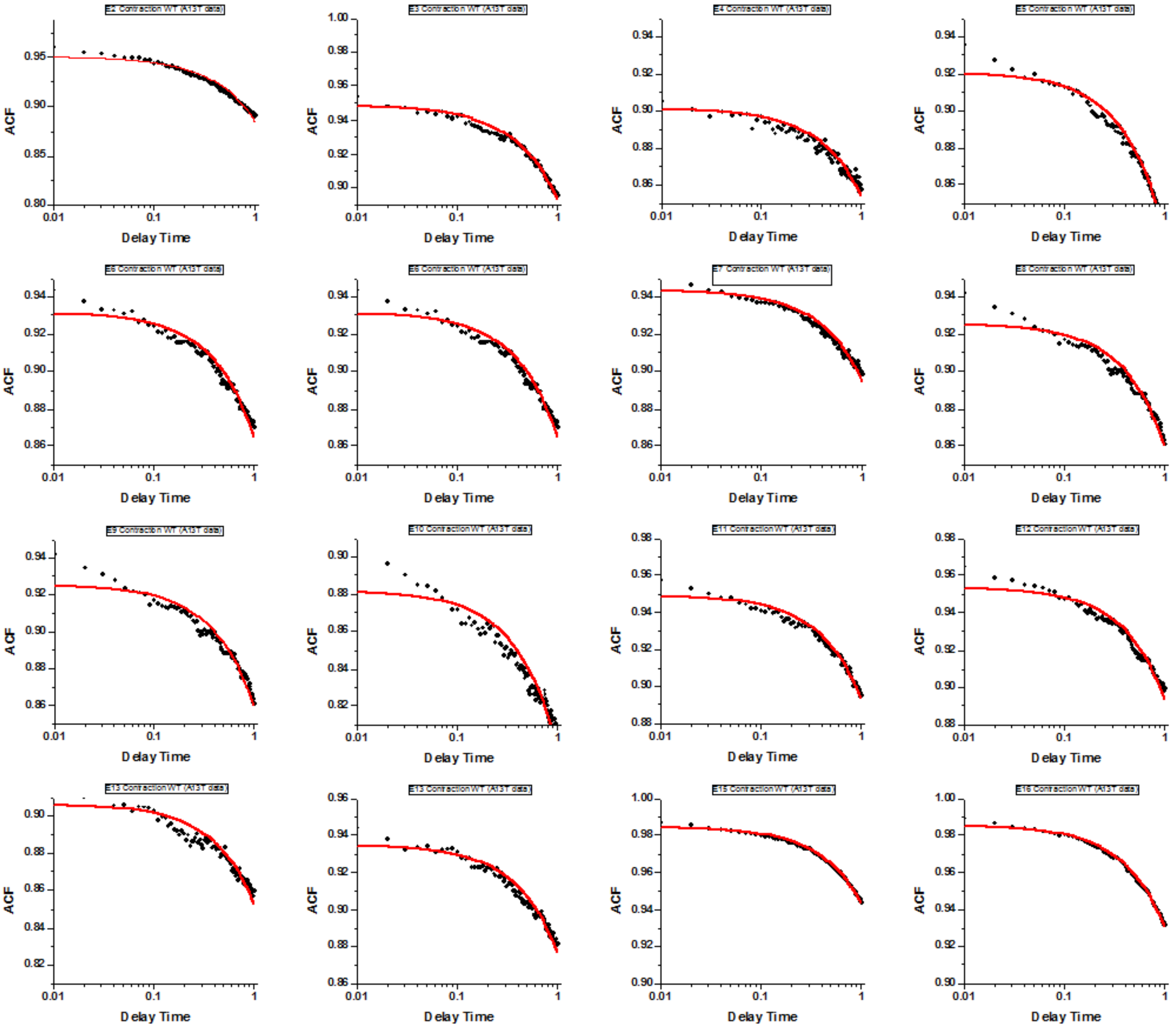

Fig. 3S cntd.

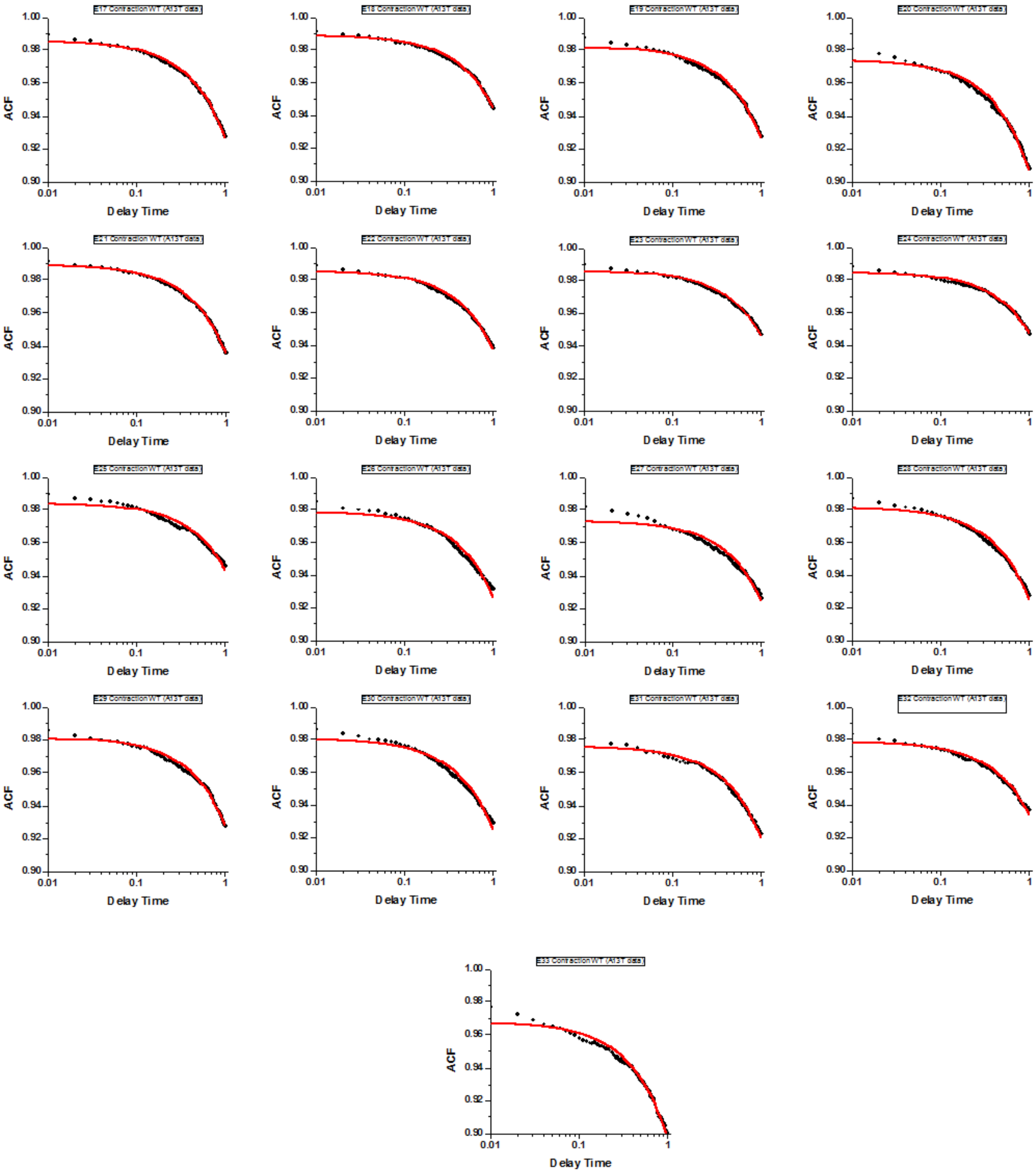
E17 Contraction WT (A13T data)
E18 Contraction WT (A13T data)
E19 Contraction WT (A13T data)
E20 Contraction WT (A13T data)
E21 Contraction WT (A13T data)
E22 Contraction WT (A13T data)
E23 Contraction WT (A13T data)
E24 Contraction WT (A13T data)
E25 Contraction WT (A13T data)
E26 Contraction WT (A13T data)
E27 Contraction WT (A13T data)
E28 Contraction WT (A13T data)
E29 Contraction WT (A13T data)
E30 Contraction WT (A13T data)
E31 Contraction WT (A13T data)
E32 Contraction WT (A13T data)
E33 Contraction WT (A13T data)
ACF
Delay Time

Supplemental Figure 4S Traces of normalized autocorrelation functions of all 33 experiments of polarization of fluorescence of contracting myofibril prepared from the left ventricle of **Tg-A13T** mouse. Circles are experimental data. A red line is the fit to the equation for ACF (equation of Fig. 3S). The fact that the correlation decays in time indicates that the orientation of absorption/emission dipoles change in time. The fact that ACF decays to a value >0 is due to the fact that mean polarization was non-zero. Delay time is in seconds. ACF's judged by AR^2 to be nonstandard are outlined in blue. The nonstandard ACF's are outlined in color.

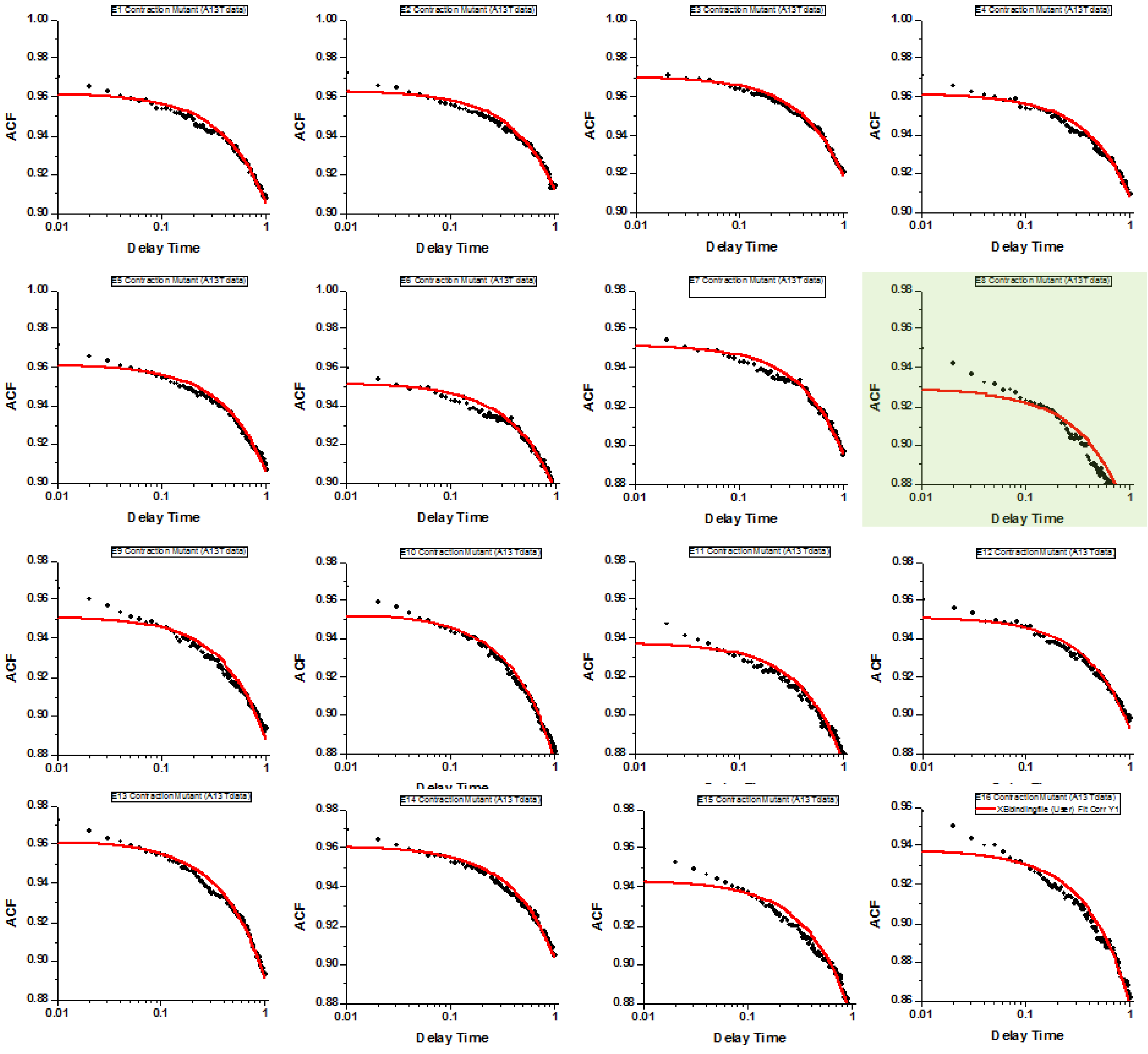

Supplemental Figure 4S – cntd

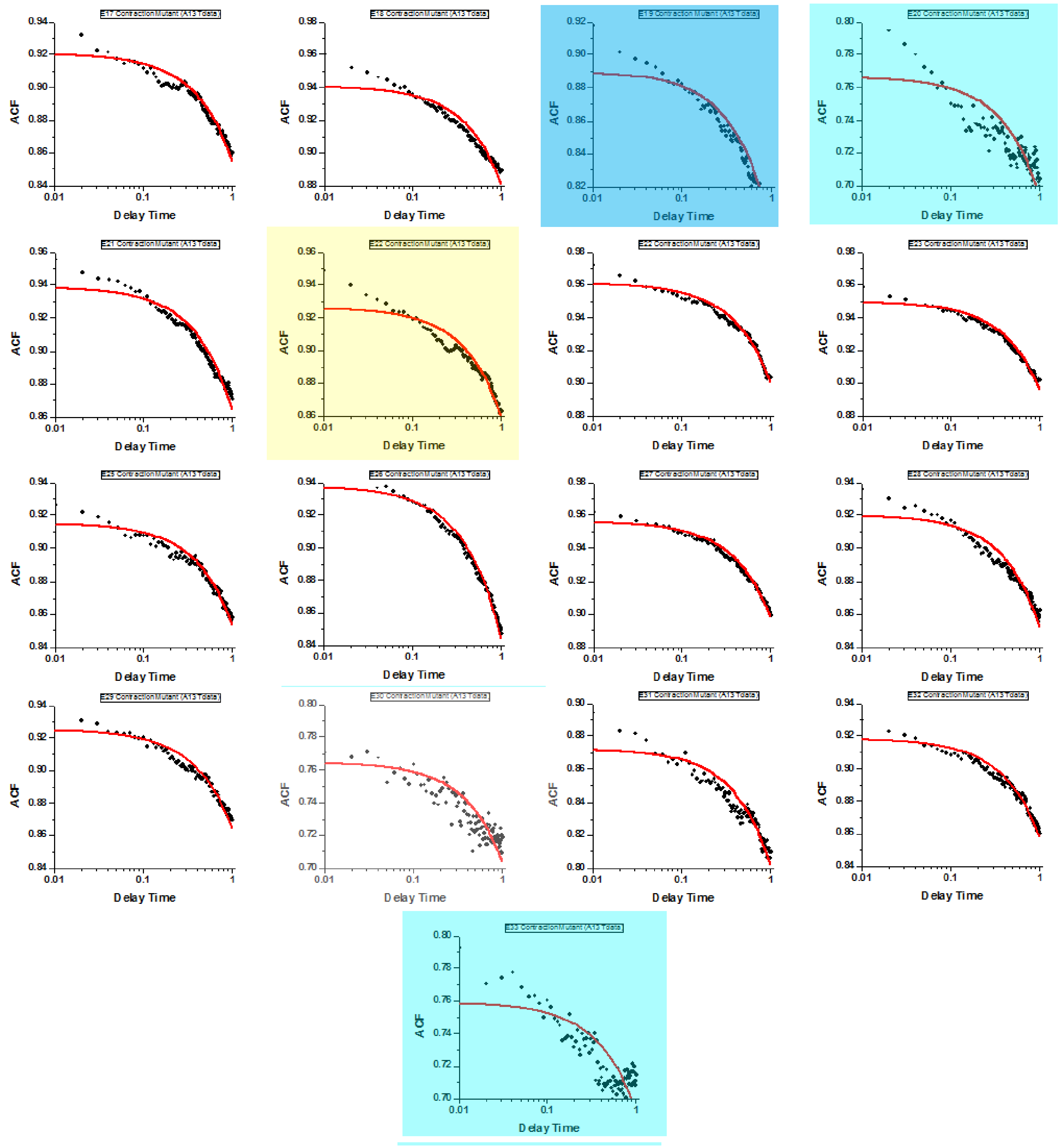

Supplemental Figure 5S. Histograms of all 32 experiments of polarization of fluorescence of contracting myofibrils prepared from the left ventricle of **Tg-WT** mouse. A red line is the fit to a Gaussian curve $y=a\ exp[-0.5(x-x_o/b)^2]$. Histograms judged to be nonstandard are outlined in blue. One experiment was omitted.

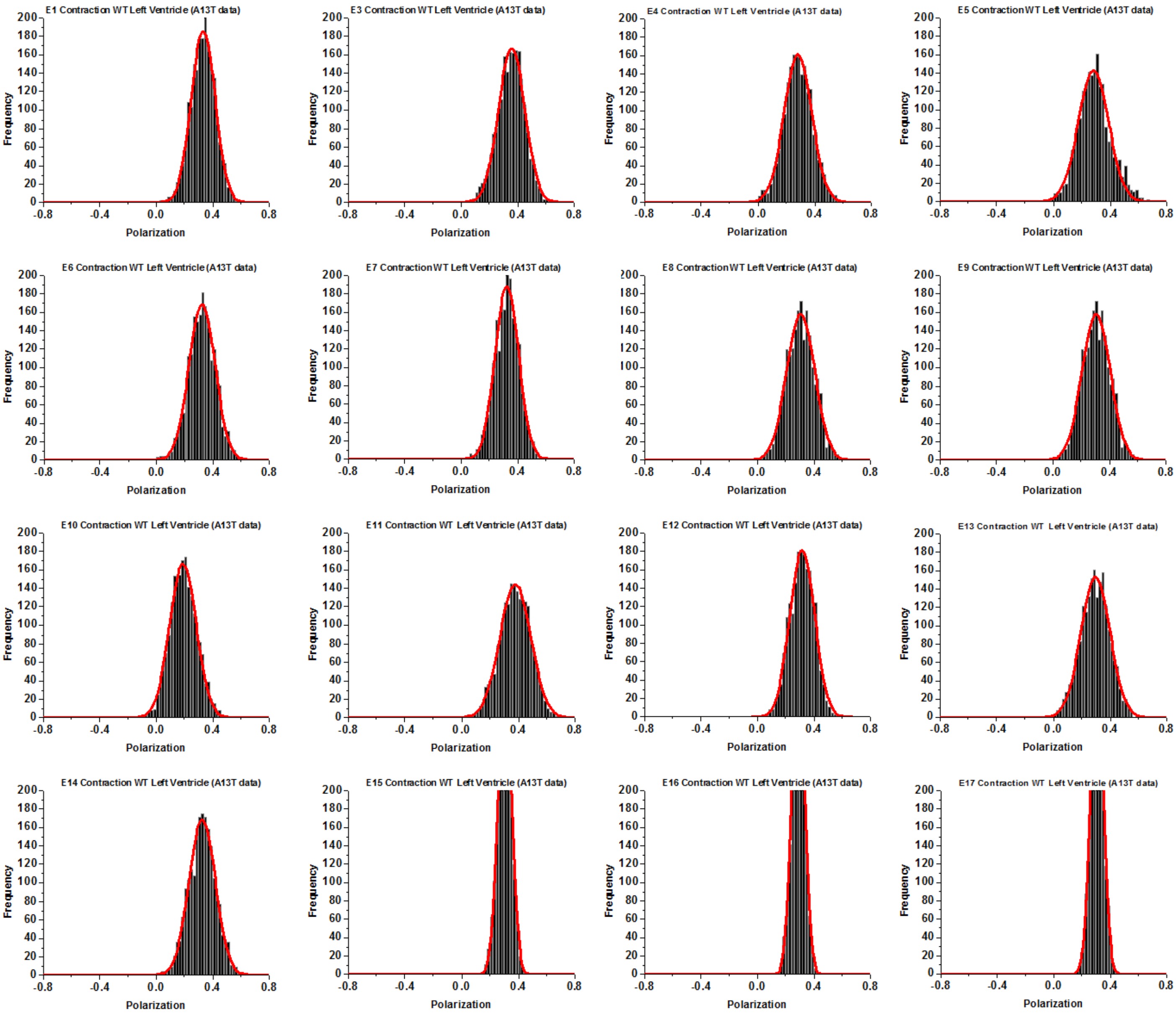

Supplemental Fig. 5S - cntd

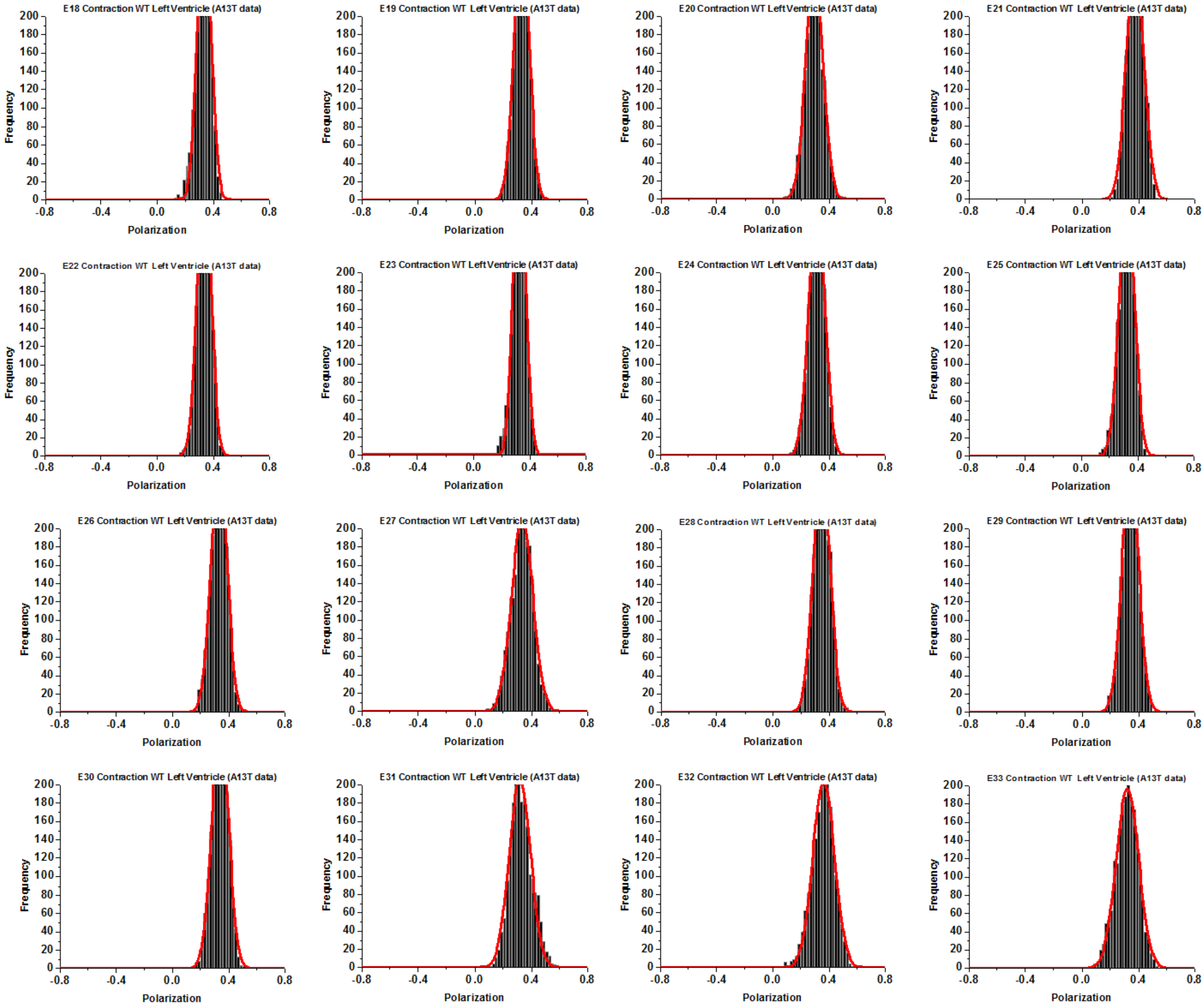

Supplemental Figure 6S. Histograms of all 33 experiments of polarization of fluorescence of contracting myofibrils prepared from the left ventricle of **Tg-A13T** mouse. A red line is the fit to a Gaussian curve y=a exp[-0.5(x-x_o/b)^2] . Histograms judged to be nonstandard are outlined in blue.

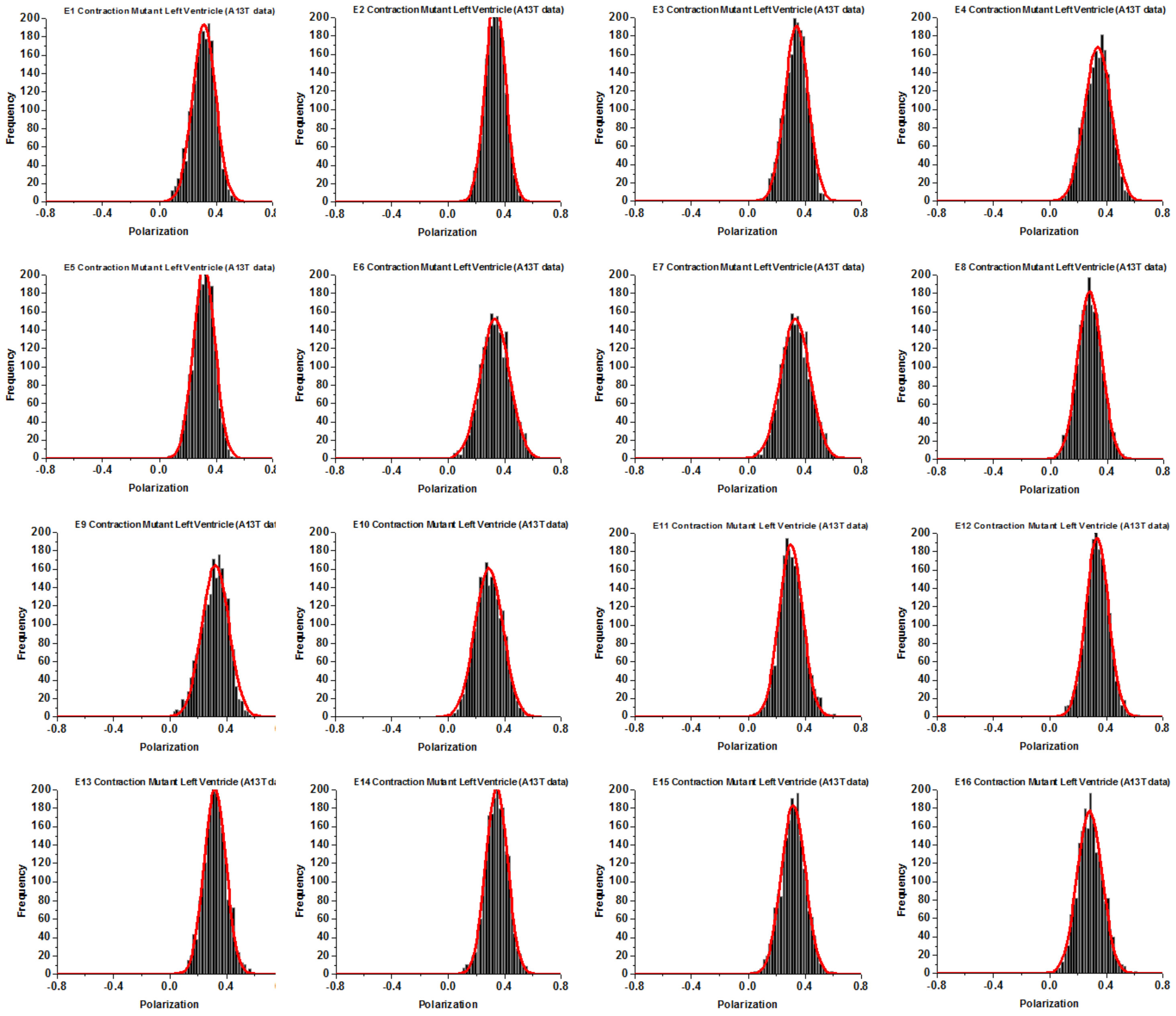

Supplemental Fig. 6S - cntd

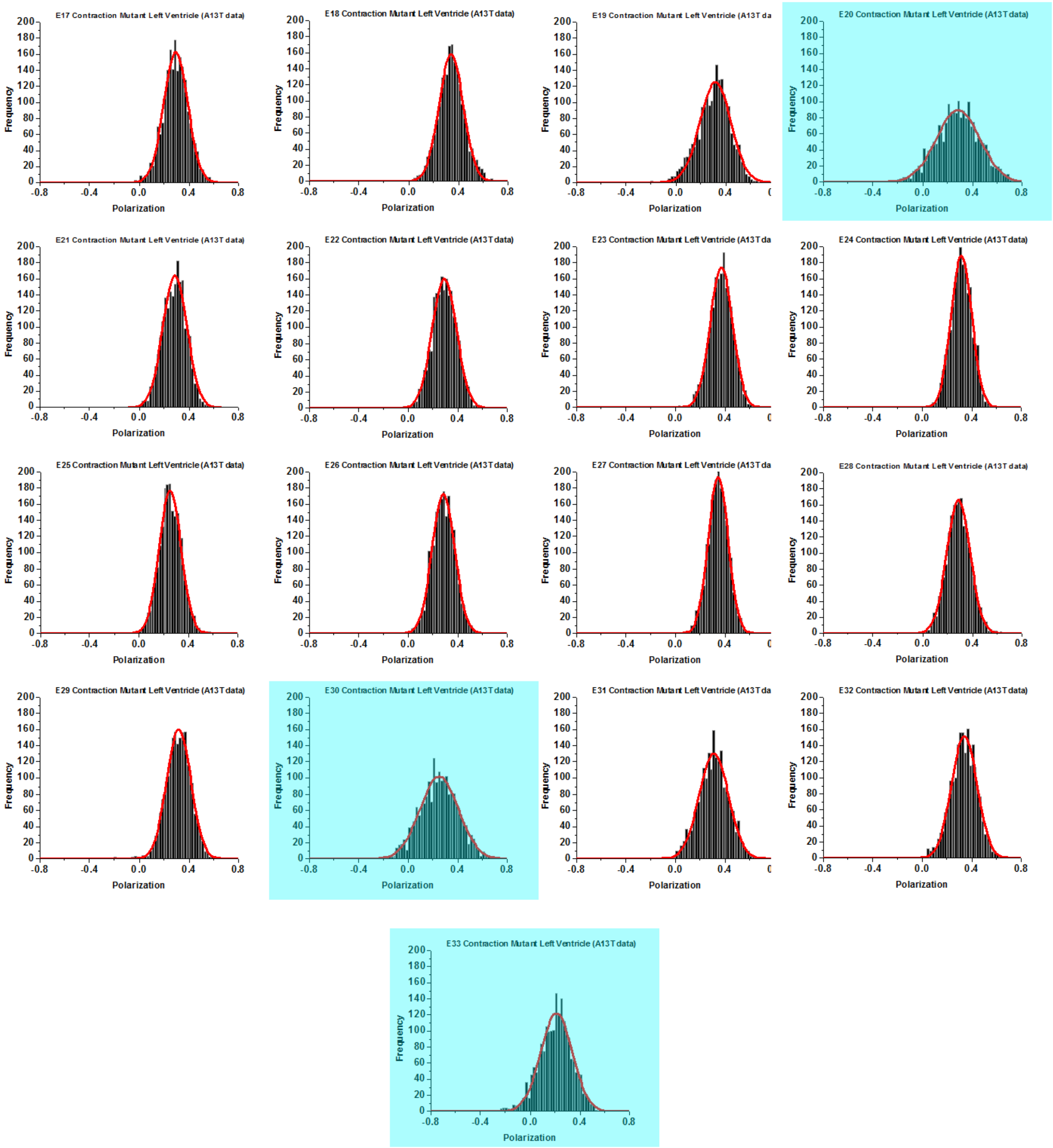

REFERENCES

1. Magde, D., E.L. Elson, and W.W. Webb, *Fluorescence correlation spectroscopy. II. An experimental realization.* Biopolymers, 1974. **13**(1): p. 29-61.
2. Elson, E.L., *Quick tour of fluorescence correlation spectroscopy from its inception.* J Biomed Opt., 2004. **9**(5): p. 857-64.
